# Cost of Providing Quality Cancer Care at the Butaro Cancer Center of Excellence in Rwanda

**DOI:** 10.1200/JGO.17.00003

**Published:** 2017-07-17

**Authors:** Claire Neal, Christian Rusangwa, Ryan Borg, Neo Tapela, Jean Claude Mugunga, Natalie Pritchett, Cyprien Shyirambere, Elisephan Ntakirutimana, Paul H. Park, Lawrence N. Shulman, Tharcisse Mpunga

**Affiliations:** **Claire Neal**, University of North Carolina Gillings School of Global Public Health, University of North Carolina, Chapel Hill, NC; **Christian Rusangwa**, **Ryan Borg**, **Natalie Pritchett**, **Cyprien Shyirambere**, **Elisephan Ntakirutimana**, and **Paul H. Park**, Partners In Health/Inshuti Mu Buzima, Kigali; **Tharcisse Mpunga**, Ministry of Health, Butaro, Rwanda; **Neo Tapela** and **Paul H. Park**, Brigham and Women’s Hospital; **Jean Claude Mugunga**, Partners In Health, Boston, MA; and **Lawrence N. Shulman**, Abramson Cancer Center, University of Pennsylvania, Philadelphia, PA.

## Abstract

**Purpose:**

The cost of providing cancer care in low-income countries remains largely unknown, which creates a significant barrier to effective planning and resource allocation. This study examines the cost of providing comprehensive cancer care at the Butaro Cancer Center of Excellence (BCCOE) in Rwanda.

**Methods:**

A retrospective costing analysis was conducted from the provider perspective by using secondary data from the administrative systems of the BCCOE. We identified the start-up funds necessary to begin initial implementation and determined the fiscal year 2013-2014 operating cost of the cancer program, including capital expenditures and fixed and variable costs.

**Results:**

A total of $556,105 US dollars was assessed as necessary start-up funding to implement the program. The annual operating cost of the cancer program was found to be $957,203 US dollars. Radiotherapy, labor, and chemotherapy were the most significant cost drivers. Radiotherapy services, which require sending patients out of country because there are no radiation units in Rwanda, comprised 25% of program costs, labor accounted for 21%, and chemotherapy, supportive medications, and consumables accounted for 15%. Overhead, training, computed tomography scans, surgeries, blood products, pathology, and social services accounted for less than 10% of the total.

**Conclusion:**

This study is one of the first to examine operating costs for implementing a cancer center in a low-income country. Having a strong commitment to cancer care, adapting clinical protocols to the local setting, shifting tasks, and creating collaborative partnerships make it possible for BCCOE to provide quality cancer care at a fraction of the cost seen in middle- and high-income countries, which has saved many lives and improved survival. Not all therapies, though, were available because of limited financial resources.

## INTRODUCTION

The global cancer burden is expected to grow to more than 21 million new cancer cases by 2030.^1^ Much of this burden will be borne by populations living in low- and middle-income countries. In 1970, only 15% of new cancer cases occurred in the developing world.^2^ Currently, more than half of new cancer cases and almost two thirds of cancer deaths occur in low- and middle-income countries.^1^ Health care systems in low-income countries are often ill-equipped to handle this growing burden, and the cost of providing care in these settings is not well understood.

The Butaro Hospital in the northern Burera district of Rwanda is home to the Butaro Cancer Center of Excellence (BCCOE). BCCOE is the referral center for cancer care for both the district and the country, but patients from surrounding countries and the region also arrive seeking care. In July 2012, BCCOE was inaugurated to offer preventive care, pathology-based diagnosis, staging, chemotherapy, referral for radiotherapy, follow-up, and palliative care, as well as psychosocial and practical support such as mental health and social work services, food packages, transportation assistance, and home visits.^3-5^ Imaging and surgical services that are not currently available at BCCOE are provided at referral hospitals. Cancer services (including chemotherapy and radiation therapy) are provided free of charge regardless of the patient’s ability to pay.

There are currently approximately .05 doctors per 1,000 residents in Rwanda.^5^ In 2010, there was one oncologist for the entire population of 11 million people and no pediatric oncologists.^5^ By comparison, the United States has approximately 3.8 oncologists per 100,000 people.^6^ As a result, care at the BCCOE is provided by non-oncologists, including general practitioners, internists, and pediatricians with special training in oncology. Partners in Health (PIH) collaborates with the Dana-Farber Cancer Institute to provide care and mentorship to providers at PIH-supported sites in Rwanda. Local nurses provide chemotherapy and are mentored by visiting oncology nurses from the Dana-Farber Cancer Institute. The Rwanda-based providers are supported by US-based oncologists with weekly structured phone calls and additional communication as necessary.

Although new cancer programs like BCCOE are being established in low-income countries, questions regarding the affordability of cancer care continue to create barriers to the expansion of care. The field has made some strides in recognizing the costs of inaction in terms of lost productivity, ties to poverty, and burden on the health care system.^6a^ However, models for delivering care are still relatively new, and the costs of delivering high-quality cancer care in low-resource settings have not yet been well defined.^7^ To evaluate the cost of providing comprehensive cancer care in a low-income country, the project used a microcosting approach to calculate an annual operating cost for the cancer program at the BCCOE in Rwanda.

## METHODS

We conducted a retrospective costing analysis from the provider point of view by using secondary data from the administrative systems of the Butaro Hospital. Costs were assessed during the 2014 fiscal year (July 1, 2013, to June 30, 2014) through direct measurement. Relevant activities and resources were identified and prices were assigned. Each of the following sources provided data for the study:

- Butaro Hospital Balance Sheet for the last complete fiscal year (July 2013-July 2014)
- PIH Rwanda Noncommunicable Disease FY14 Program Balance Sheet (July 2013-June 2014)
- PIH Burera District Budget
- PIH Procurement/Pharmacy Invoices
- Program data on patients diagnosed and treated between July 2013 and June 2014 in each category of disease
- 2012 Rwandan National Reference for Rates and Hospital Centers

All costs paid in Rwandan francs were converted to US dollars by using the conversion rate at the time, if it was available, or the median 2014 exchange rate of 674 Rwandan francs to 1 US dollar (USD) if a specific rate was not available. The methodology distinguishes between two different cost categories: fixed costs and variable costs.

### Fixed Costs

Fixed costs are those that remain constant despite the number of patients treated. Examples of fixed costs within the Butaro Hospital budget are electricity and utilities, repairs and maintenance, program costs, and expenses.

Start-up costs were separated from annualized overhead costs to allow for easier program planning and future cost predictions. This included the cost of renovating the hospital to expand and repair the cancer center, an ambulance purchase, National Baseline Cancer Care training, and the equipment and supply purchases necessary to equip the pathology laboratory and ward. Equipment for the pathology laboratory included all equipment necessary to run the laboratory, such as microtomes, tissue processors, cameras, and microscopes. Training costs included those related to the National Baseline Cancer Care training. This initiative, supported by GlaxoSmithKline, trained nurses and physicians across the country to give them foundational knowledge about cancer, its epidemiology, and available treatments, as well as the main cancers and treatment priorities in Rwanda. As a train-the-trainer initiative, limited training costs are ongoing; however, the bulk of the training cost was a one-time expense intended to foster a robust referral network and early diagnosis system for the country.

All one-time, nonrecurrent capital expenditures associated with the BCCOE were identified, including original construction costs of the hospital space, renovation costs of the hospital to prepare the cancer center, and equipment and supply purchases for the pathology laboratory and ward. For each cost, we included the actual price paid by the program, which included items that were purchased at a significant discount. Fixed-cost categories were identified for services shared with the hospital, and a percentage of the total was allocated to the cancer program on the basis of square footage, with the exception of equipment maintenance and repair, which was allocated on the basis of the equipment specific to the cancer ward out of the total equipment for the hospital. The totals for these categories were added to the costs directly attributable to the cancer program for a total fixed cost of the cancer program. All costs associated with overhead were annualized.

### Variable Costs

Variable costs change with patient volume and include chemotherapy drug costs, supportive medication costs, labor costs, laboratory tests, and social support.

#### Medications and consumables.

All purchases and emergency orders for chemotherapy medications, supportive medications, and consumables related to the oncology program were totaled for the fiscal year 2013-2014. Non-chemotherapy–related medications and consumables (such as paracetamol) were estimated on the basis of quarterly supply orders and then allocated to the cancer program on the basis of the proportion of beds.

#### Radiotherapy.

Because there are no radiotherapy capabilities in Rwanda, patients in need of radiotherapy were sent to Uganda. All costs related to treatment in Uganda, including transport, medical bills, lodging, and meals were totaled for the year.

#### Laboratory tests and surgeries.

Services provided to the oncology program from the University Central Hospital of Kigali and the Rwanda Military Hospital, including laboratory tests such as prostate-specific antigen, thyroid-stimulating hormone, and beta-human chorionic gonadotropin (pregnancy test), computed tomography (CT) scans, and surgeries were estimated on the basis of invoice totals from the 2015 calendar year.

#### Blood products.

Blood product orders placed in 2015 were used to determine a monthly average for estimating the total cost of blood products during the study period.

#### Social support.

Loss to follow-up rates can be high in low-income countries, so social support is often necessary to ensure that patients can complete a full course of cancer treatment. Relevant costs included transportation to and from treatment, mental health and social work services, home visits, new on-site housing for ambulatory patients with cancer, and assistance with burial costs. All relevant social support costs from both the District Ministry of Health and the PIH oncology budgets were totaled for the year.

#### Pathology.

The Butaro Hospital anatomic and clinical pathology laboratory processes almost 2,000 samples each year. A diagnosis of cancer is determined through the pathology laboratory in which samples are prepared and processed. In 2014, a telepathology system was initiated that allowed for increased diagnostic capacity, decreased turnaround time, and improved training.^8,9^ US-based volunteer laboratory technicians and pathologists provided mentorship both in Rwanda and remotely. No local pathologists were available during this study to provide diagnoses. As a result, prepared samples were either shipped to Boston for review at the Brigham and Women’s Hospital or were uploaded for remote pathology review using the telepathology system. During the study, the diagnostic services were provided by Brigham and Women’s Hospital free of charge to the program and are therefore not included in the analysis. Costs to the program for the development of pathology services were captured in the PIH oncology budget and included items such as shipment of samples to Boston, training and travel costs for pathology laboratory technicians, and customs and duties for reagents.

#### Labor.

All paid staff who supported the oncology program were identified, including cashiers, anesthetists, an archivist, a stock manager, plumbers, data managers, drivers, laboratory technicians, electricians, physicians, nurses, cleaning staff, social workers, and a nutritionist. The annual salary for each position was assessed, including allowances for transportation and accommodation. Additional amounts for Rwanda’s Medical Insurance Agency and the Rwanda Social Security Board were included. Provision of care is significantly bolstered by doctors who volunteer their time to the cancer program. Because this labor is readily available free of charge to the cancer program from multiple sources, the cost for volunteer labor was not included in this analysis. Table 1 provides a summary of the oncology program cost categories and the significance of each category.

**Table 1:**
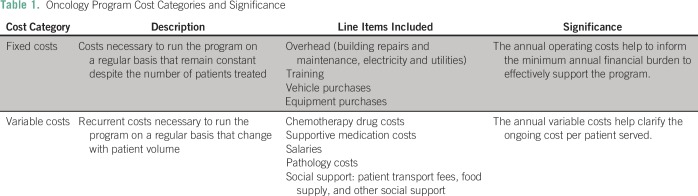
Oncology Program Cost Categories and Significance

## RESULTS

We determined that $556,105 (USD) in start-up funds was necessary to begin initial implementation of the BCCOE within the existing infrastructure. Details are provided in Table 2. Costs included the cost of renovating the hospital to expand and repair the cancer center, purchase of an ambulance, providing a national baseline cancer training, and purchasing the equipment and supplies necessary to equip the pathology laboratory and clinical cancer ward.

**Table 2:**
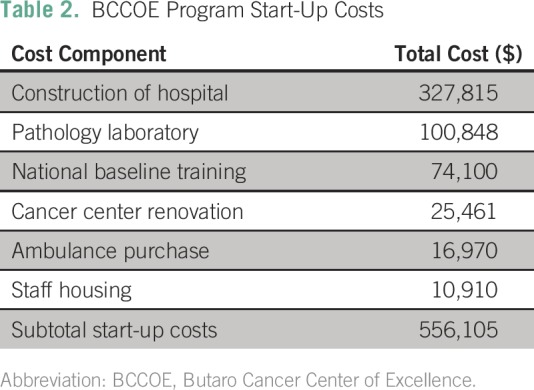
BCCOE Program Start-Up Costs

The annual operating cost of the cancer program at the BCCOE was determined to be $957,203 USD (Table 3). Between July 2013 and June 2014, 2,576 oncology patients were seen at the BCCOE; 1,290 patients were newly enrolled.

**Table 3:**
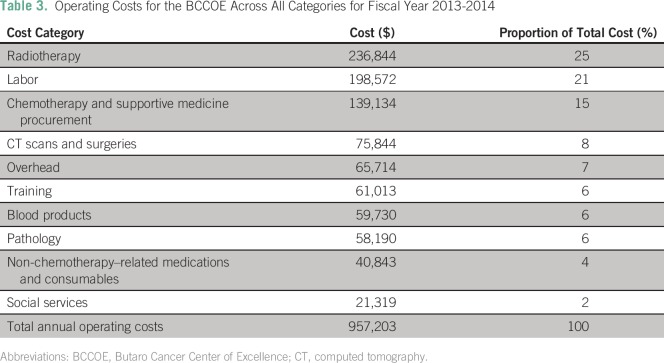
Operating Costs for the BCCOE Across All Categories for Fiscal Year 2013-2014

**Fig 1:**
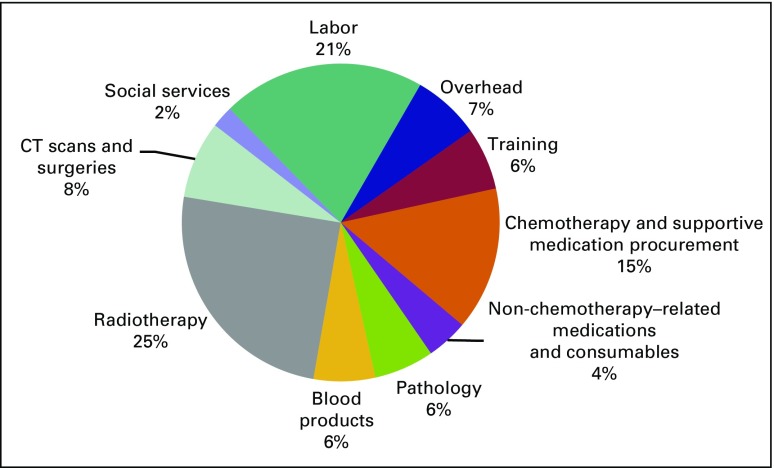
Total annual program operating costs for the Butaro Cancer Center of Excellence by category. CT, computed tomography.

Chemotherapy, radiotherapy, and labor were the most significant cost drivers for the program. Despite the relatively low number of patients sent to Uganda, radiotherapy services were the most significant cost driver, comprising 25% of program costs. It should be noted that only a small percentage of patients who would have benefited from radiotherapy were sent to Uganda for this treatment because of cost constraints. Labor was the second largest cost driver, accounting for 21% of the overall cost, followed by chemotherapy and supportive medications and consumables at 15%. Overhead, training, CT scans and surgeries, blood products, non-chemotherapy–related medications and supplies, pathology, and social services each accounted for less than 10% of the total. Figure 1 presents the proportions of cost by category.

## DISCUSSION

This study is one of the first to examine start-up and annual operating costs for implementation of a cancer center in a low-income country. The results will be useful in considering costs and cost drivers when establishing new cancer facilities in Rwanda and beyond.

There are a number of factors that contribute to the ability of programs like BCCOE based in low-income countries to provide high-quality effective cancer care at lower costs than middle- and high-income countries can. Partnership was a critical component in establishing the cancer center, particularly in the areas of labor, staff training, and the development of pathology capabilities. The establishment of strong partnerships allowed for cost sharing and volunteer labor to augment services while local capacity was built. As with many developing countries, the cost of labor for the cancer center is significantly less expensive than in more economically developed countries. In addition, Rwanda uses a task-shifting model to allow generalists (pediatricians, internists, and general practitioners) to provide care with structured support from US-based oncologists. This further reduces the cost of providing care. Labor costs for the entire cancer center (including all positions from physicians, nurses, and nutritionists to custodians and cashiers) is less than the average annual salary of $246,526 USD for one oncologist in the United States.^10^ In addition, chemotherapy regimens that are effective in high-income countries are not necessarily the same treatment strategies needed in low-income settings. Locally adapted protocols that minimize toxicity and length of stay in the hospital (and are therefore less expensive than those followed in high-income countries) have been shown to cure a significant number of children when applied with appropriate social support in low-resource settings.^11,12^ Some costly systemic therapies such as trastuzumab and rituximab were not purchased because of high costs and limited financial resources. If all patients with accepted indications for these drugs were treated with them, the cost of systemic therapy would have been greater. In addition, imatinib for the treatment of chronic myeloid leukemia and gastrointestinal stromal tumors was donated free of charge from the Max Foundation and the Gleevec International Patient Assistance Program. Finally, expensive treatment modalities such as radiotherapy, which are common in higher-income settings, are limited to those most in need when resources are scarce. In the case of radiotherapy, only a fraction of patients who would have benefitted from this treatment were sent to Uganda because of limited financial resources. If all patients with accepted indications for radiation therapy were sent for treatment, the cost would have been much greater. These essential adaptations allow BCCOE to provide high-quality care within available resources.

The costs enumerated for radiotherapy services make a strong case for the development of local radiotherapy capacity in Rwanda. Radiotherapy has been estimated to lead to a cure in 40% of patients “compared to 49% of patients being cured by surgery, and 11% of patients by systemic treatments.”^13^ Radiotherapy capacity is extremely limited in low-income countries, with no radiotherapy units at all located in Rwanda. The initial investment for a basic radiotherapy clinic with two megavoltage units^14^ is estimated to be between $5 million USD and $6 million USD. This estimate includes the building, equipment, and human resources necessary to run the equipment. This is a hefty up-front investment, but when amortized over the life of the clinic, the resultant cost is expected to be between $250,000 USD and $400,000 USD annually. The annual cost of providing radiotherapy services through collaboration with the Mulago Hospital in Uganda was estimated to be between $237,000 USD and $308,000 USD. Rwanda’s existing investment in radiotherapy is already close to the estimated annual investment for local capacity. With local radiotherapy services, a much higher number of patients could receive treatment than are currently receiving care for the existing investment. However, it will be important to consider additional ongoing costs such as maintenance, quality control, and staff training when evaluating this investment.

This study is limited by the perspective of the analysis. Our focus was on capturing the costs of implementing an effective cancer program and did not include the larger societal costs of treatment, such as the out-of-pocket expenses of patients or lost productivity during treatment. An analysis of societal costs should include not only the time the patient spends away from school and home but also the lost productivity of the family member who must accompany the patient, who loses time away from work and other family members. The cost of treatment for the family and society as a whole can be significant but is not reflected in this study.

A number of data points were restricted or unavailable. The estimate for CT scans and surgeries was based on the total price paid by the oncology program to the primary hospitals in Kigali. This price provides a good estimate of the price paid by the implementing agency but is not useful in understanding the overall cost of the procedures from the perspective of the Ministry of Health, because the estimate does not reflect the true cost of providing these services. The costs covered by the program can represent anywhere between 10% and 100% per patient. A full review of hard-copy invoices across all cancer types from the CHUK and RMH systems was outside the scope of this study; however, this type of review would provide a better estimate of the full cost of these services. We were able to attribute chemotherapy and supportive care medications directly to the cancer program; however, the use of general medications (such as ibuprofen) or consumables unrelated to chemotherapy (such as gloves) were not associated directly with the program. We had electronic access to one quarter of stock purchases for the pharmacy for such items and extrapolated to get a yearly cost which was then attributed to the cancer program on the basis of beds. This was our best available methodology given the data, but it likely overstates the cost of these medications and consumables to the cancer program.

The BCCOE relies on volunteer labor (and particularly so during the scale-up stage of development), including volunteer physicians and nurses. Providers volunteer their time with no salary cost to the cancer program or the Ministry of Health. The local replacement value of these volunteer salaries was not included in the analysis of program costs. As the programs shifts to the use of local labor, the cost of providing salaries for these services locally will need to be added to the analysis.

In conclusion, the example of the BCCOE provides a reason for optimism in the fight against cancer. The results make a strong case for the affordability of providing cancer care in a low-income country, when such services are provided in a public hospital setting. Although treatment options are still not comparable to those in resource-rich settings because some costly systemic therapies are not provided and because some patients who may benefit from radiation do not receive it, many patients with cancer are now receiving quality treatment who otherwise would not have had other options. Greater investment in cancer care would allow for further development of the program and for saving more lives. By combining a strong commitment to cancer care, adapting protocols to the local setting, shifting tasks, and creating collaborative partnerships, BCCOE has shown that establishing an affordable, quality cancer care program in a low-income country is possible.
